# Cellular shape micromachined actuator ribbons

**DOI:** 10.1038/s41378-022-00421-y

**Published:** 2022-08-03

**Authors:** Amin Abbasalipour, Prithviraj Palit, Sepehr Sheikhlari, Siavash Pakdelian, Siavash Pourkamali

**Affiliations:** 1 Department of Electrical and Computer Engineering, University of Texas at Dallas, Richardson, TX USA; 2 Department of Electrical and Computer Engineering, University of Massachusetts, Lowell, MA USA

**Keywords:** Electrical and electronic engineering, NEMS

## Abstract

This work presents a new class of micromachined electrostatic actuators capable of producing output force and displacement unprecedented for MEMS electrostatic actuators. The actuators feature submicron high aspect ratio transduction gaps lined up in two-dimensional arrays. Such an arrangement of microscale actuator cells allows the addition of force and displacements of a large number of cells (up to 7600 in one demonstrated array), leading to displacements ranging in the hundreds of microns and several gram forces of axial force. For 50 µm thick actuators with horizontal dimensions in the 1–4 millimeter range, an out-of-plane displacement of up to 678 µm at 46 V, a bending moment of up to 2.0 µNm, i.e., 0.08 N (~8 gram-force) of axial force over a 50 µm by 2 mm cross-sectional area of the actuator (800 kPa of electrostatically generated stress), and an energy density (mechanical work output per stroke per volume) up to 1.42 mJ/cm^3^ was demonstrated for the actuators.

## Introduction

Electromechanical actuators that convert electrical energy into mechanical force or motion are an integral part of any electrically powered system with moving parts. Examples of such systems with subcentimeter dimensions include microrobots^1–4^, precision positioning systems^5–8^, optical systems^9–11^ and medical devices^12–16^. For such applications, the actuator must provide displacements ranging from tens of micrometers to millimeters and supply forces in the mN range. Batch-fabricated micromachined (Microelectromechanical system) actuators can provide low-cost highly integrated solutions for these applications. However, none of the existing micromachined electrostatic actuators can meet the large energy output requirements. Transduction mechanisms commonly used in micromachined actuators include electrostatic, piezoelectric, electrothermal, and electromagnetic mechanisms. Electromagnetic transduction, which is the main means of electromechanical energy conversion in macroscale systems, has been conventionally used in smaller scale systems. These actuators, such as voice coil motors (VCM), can produce relatively large force and displacement and are widely used for autofocus (AF) and optical image stabilization (OIS) in compact camera modules in modern consumer electronics (smartphones, tablets, etc.)^17–19^. However, the need for high-turn coils and magnets makes such actuators difficult to miniaturize and batch fabricate by micromachining. They are also not power efficient due to the need for significant current flow in the coils, potentially even when the actuator is not moving. Piezoelectric actuators, on the other hand, are very power efficient and provide a high output force, but a very small stroke necessitates aggressive leverage mechanisms to reach adequate displacements at the cost of lowering the force^20–22^. Furthermore, since the inclusion of piezoelectric materials in micromachining processes is mainly limited to thin films, reaching a large overall actuator active layer size and thus a high energy output is very challenging. Finally, electrothermal microactuators utilizing thermal expansion and contraction of heated elements can produce large force and displacement (with leverage), but their high-power consumption is prohibitive for most applications^23,24^.

Another type of micro/millimeter-scale actuator is shape memory alloy (SMA) actuators, which provide a large range of displacement and a high work/weight ratio. It has been recently demonstrated that an SMA NiTi actuator fabricated with femtosecond laser ablation provides a maximum stroke of 800 µm and yields a maximum actuator force of 1150 µN^25^. However, due to their relatively long cooling time, SMA actuators have small bandwidths and low operating frequencies. Furthermore, NiTi, which is the most commonly used SMA due to properties such as a large shape memory effect and relatively high long-term stability^26^, is still challenging to integrate into microsystems because of its difficulty in connecting to other materials^27^.

Electrostatic actuators, on the other hand, are highly power efficient and compatible with semiconductor manufacturing processes. Electrostatic transducers, while rarely used for power conversion in macroscale systems, are widely used in microelectromechanical systems (MEMS). Examples include vibratory gyroscopes^28,29^, accelerometers^30–32^, MEMS silicon oscillators^33–35^, and capacitive micromachined ultrasonic transducers (CMUT)^36,37^. Electrostatic transducers are suitable for microscale systems because their narrow air gaps, with sizes ranging in microns, can withstand much higher electric fields than larger air gaps without breaking down. According to Paschen’s law^38,39^, air gaps ranging from several microns and below can withstand extremely large electric fields (hundreds of MV/m as opposed to 3 MV/m for millimeter-scale gaps) without breaking down^40,41^. The stored energy density in these electric fields can therefore even surpass 1.0 J/cm^3^, i.e., a 20X higher energy density than in animal muscles. Electrostatic actuators with air gaps can also provide displacements ranging in microns without the need for leverage mechanisms. This, along with the ability to expand in three dimensions (into the bulk of a substrate as opposed to piezoelectric thin films), makes electrostatic actuators an interesting option for achieving high output actuation.

Nonetheless, reaching a displacement range of hundreds of microns with an output force in the mN range for micromachined electrostatic actuators and competing with voice coil motors is challenging. The displacement of a parallel plate electrostatic actuator is fundamentally limited to the air gap size. Increasing the air gap leads to a significantly lower output force or higher operating voltage, while the considerable breakdown electric field advantage starts to disappear for air gaps above 10 µm wide. Comb-drive actuators can bypass this limitation to some extent but offer much lower energy densities, as the transduction only occurs by the fringing fields at the tip of the electrodes as opposed to the whole air gap area. Inchworm motors represent an interesting approach to maintain the high energy density of parallel plate actuators while bypassing the displacement range limitation via periodic back-and-forth motions along with an integrated locking mechanism^42^. In this manner, displacement from several strokes of the actuator can add up to reach a large overall displacement. A force density of approximately 2 mN/mm^2^ for operation at 110 V and a maximum in-plane displacement of 124 μm^43^ have been demonstrated for inchworm motors. The wear and tear of the lock-in mechanism, possibility from stiction, and limited speed due to the required periodic back-and-forth motion are among the major limitations for inchworm motors.

An out-of-plane displacement of 28 µm at 80 V has been demonstrated for a piston style electrostatic actuator^44^. This actuator is essentially a vertically formed large (mm-scale) comb-drive actuator formed via wafer bonding, hence the low output energy density of 0.178 µJ/cm^3^. Another recent work based on the active bending of cantilevers was focused on obtaining large deflections. The actuator class was fabricated in a CMOS-compatible process that allows high deflections even with small electrode separation. The electrode separation of the fabricated actuator cells varied between 110 nm and 226 nm and gave a static deflection of 226 nm at 45 V. This actuator class allowed deflection with travel ranges widely beyond the pull-in limit, even with small electrode separation^45^. However, despite significant potential and efforts, no viable millimeter-scale actuation solutions have been offered for MEMS electrostatic actuators to date.

Inspired by the cellular structure of biological muscle tissue^46,47^, the approach proposed in this work is based on two-dimensional arrays of parallel-plate actuator cells to combine the force and displacement of individual cells, achieving an unprecedented range of stroke and output force.

The utilization of a high aspect ratio polysilicon and single crystalline silicon (HARPSS)^48^ fabrication approach allows the formation of submicron electrostatic transduction gaps within thick silicon structures. This leads to a large output energy density for the resulting actuators while maintaining relatively low actuation voltages.

The feasibility of microfabrication and the operating principle of two-dimensional arrayed electrostatic parallel-plate actuators were first presented by Abbasalipour et al.^49^. In this course of study, a new class of millimeter-scale devices with a large range of displacement and high output force is presented along with comprehensive finite element analysis (FEA) and a characterization of composite structures.

## Results

### Actuator design and operation principle

**Fig. 1:**
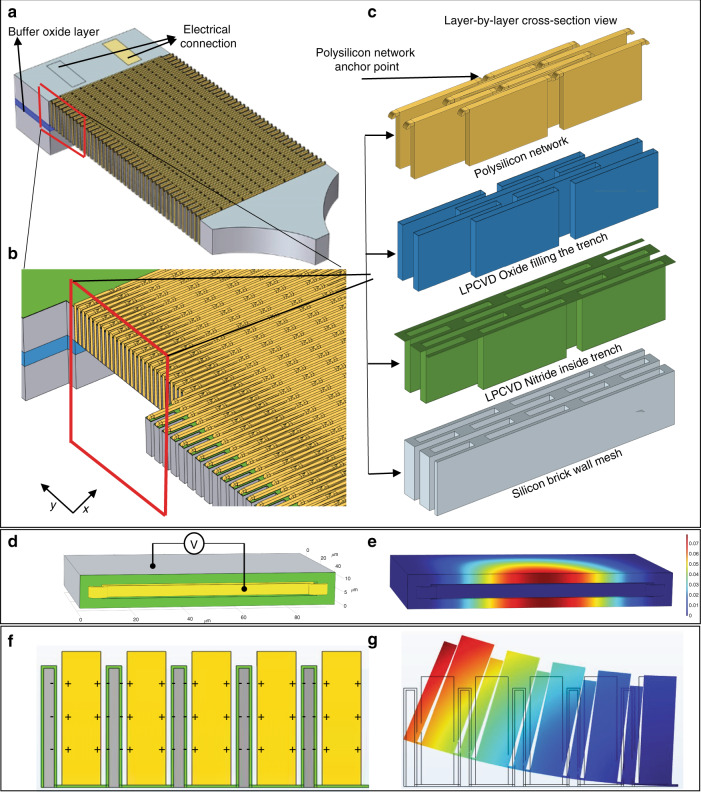
Structure of cellular shape actuator. Schematic diagrams showing (**a**) a cellular electrostatic actuator comprised of a silicon mesh with brick wall structure forming the main frame (backbone) of the actuator (grey); (**b**) zoomed view within the mesh filled by a network of electrically connected polysilicon electrodes separated from the silicon walls by transduction air gaps; (**c**) layer-by-layer structural arrangement of each individual network layer inside the actuator; (**d**, **e**) top view of single cell and the displacement profile of the cell upon application of electrostatic force, which pull the sidewalls towards each other; (**f**) lateral cross-sectional view of an array of cells and charge distribution upon the application of actuation voltage; (**g**) displacement profile of the cell array, where the lateral electrostatic force pulls the electrodes towards each other

Each cellular electrostatic actuator is composed of an arrangement of individual parallel plate actuator cells with submicron transduction air gaps in large two-dimensional suspended arrays. The force and displacement of individual microscale actuator cells integrated within the array are summed, leading to a relatively large actuation force and displacement amplitude. Figure 1a shows a schematic view of a rectangular cellular actuator array. The main skeleton (backbone) of the actuator is a crystalline silicon conductive mesh with moderate stiffness and the ability to undergo significant strain. In this manner, even though the actuators are made of hard materials, the flexible mesh can be designed to mimic the functionality and flexibility of a soft material with the desired stiffness. The openings within the mesh are filled by a network of another conductive material (polysilicon in this work) separated from the actuator walls by narrow actuation air gaps with thicknesses ranging from tens of nanometers to a few microns. Ultranarrow tall capacitive gaps with high aspect ratios are created between the frame sidewalls and the polysilicon filling the mesh openings (Fig. 1c) using a variation of the high aspect ratio polysilicon and single crystalline silicon (HARPSS) fabrication technique. The frame (or electrode) sidewalls are to be covered by a thin dielectric layer to avoid electrical shorts between electrodes and the sidewalls upon potential contact. Silicon nitride has been used in this work to cover silicon mesh sidewalls, as it is widely available and compatible with silicon micromachining. Silicon nitride is known to help with mechanical reliability by reducing the possibility of electrode and sidewall surfaces sticking upon contact^50^. The dielectric layer is also expected to increase the air gap breakdown field by blocking the flow of electrons due to field emission or tunneling. Upon application of an actuation voltage between the silicon frame and the polysilicon electrode, the attracting electrostatic force pulls the sidewalls toward the electrode in the middle, shrinking the cell width. If the structure is significantly more rigid along the axial direction on its bottom side, the electrostatic force-induced shrinkage occurs more on the softer side and will be less (or negligible) on the rigid side. This creates a bending moment in the ribbon by curling it upward. By keeping the thin film of silicon nitride on the bottom surface of the actuator, the dielectric film (silicon nitride) locks the two sidewalls on the bottom, preventing them from moving with respect to each other. Therefore, contraction of the silicon mesh only occurs on the top portion of the cells, leading to an overall bending moment curving the actuator and moving its tip upward. Each polysilicon electrode is anchored onto the silicon mesh at nodal locations (locations with close to zero deformation), i.e., the shared sidewalls between two adjacent cells in the same row, to have adequate stiffness to pull the silicon walls without becoming deformed or pulled in (Fig. 1e). The parallel plate configuration of actuator cells (as opposed to comb-drive) leads to larger forces and higher energy density. In a parallel plate electrostatic actuator, the displacement range of the movable electrode is limited to the air gap between the electrodes. A tradeoff exists between the achievable energy density and operating voltage of such actuators and the range of displacement. The array structures can bypass this limitation and enable the realization of millimeter-scale actuators with high energy density, large displacement, and moderate operating voltage. The inverse square dependence of the electrostatic force between parallel plates makes parallel plates an attractive option for actuators with ultranarrow (submicron) transduction gaps. In the case of the parallel plate cells shown, the maximum achievable deformation of individual cells is equal to the sum of the two air gaps, i.e., 2g_0_. The resulting strain in the center of each cell is the ratio of 2g_0_ to the total cell width, which includes the widths of the silicon frame sidewall, polysilicon electrode, air gaps, and sidewall dielectric films. Only half of the silicon frame sidewall width on each side of the cell is considered in the total width calculation because the cells share the sidewalls, and the outer half of the sidewall should be considered part of the adjacent cell. The resulting overall maximum strain for the whole frame is equal to the ratio of the air gap to the cell width, i.e., half as much as strain in the middle of individual cells. This is due to the arrangement of cells in a brick-wall pattern, where only half of the cells along the length of the array are aligned with each other. For example, the maximum strain for a device with a cell width of 8 µm (including silicon sidewalls, polysilicon electrodes, dielectric film, and air gaps) and a 250 nm air gap on each side of the polysilicon electrodes is 3.1%.

### Device characterization and dc test

Actuators with different sizes were fabricated using a modified HARPPS fabrication process (described in the “Materials and Methods” section) on an SOI substrate with a device layer thickness of 50 µm. The actuator array horizontal dimensions include 2 × 1 mm, 2 × 2 mm and 4 × 2 mm.

**Fig. 2:**
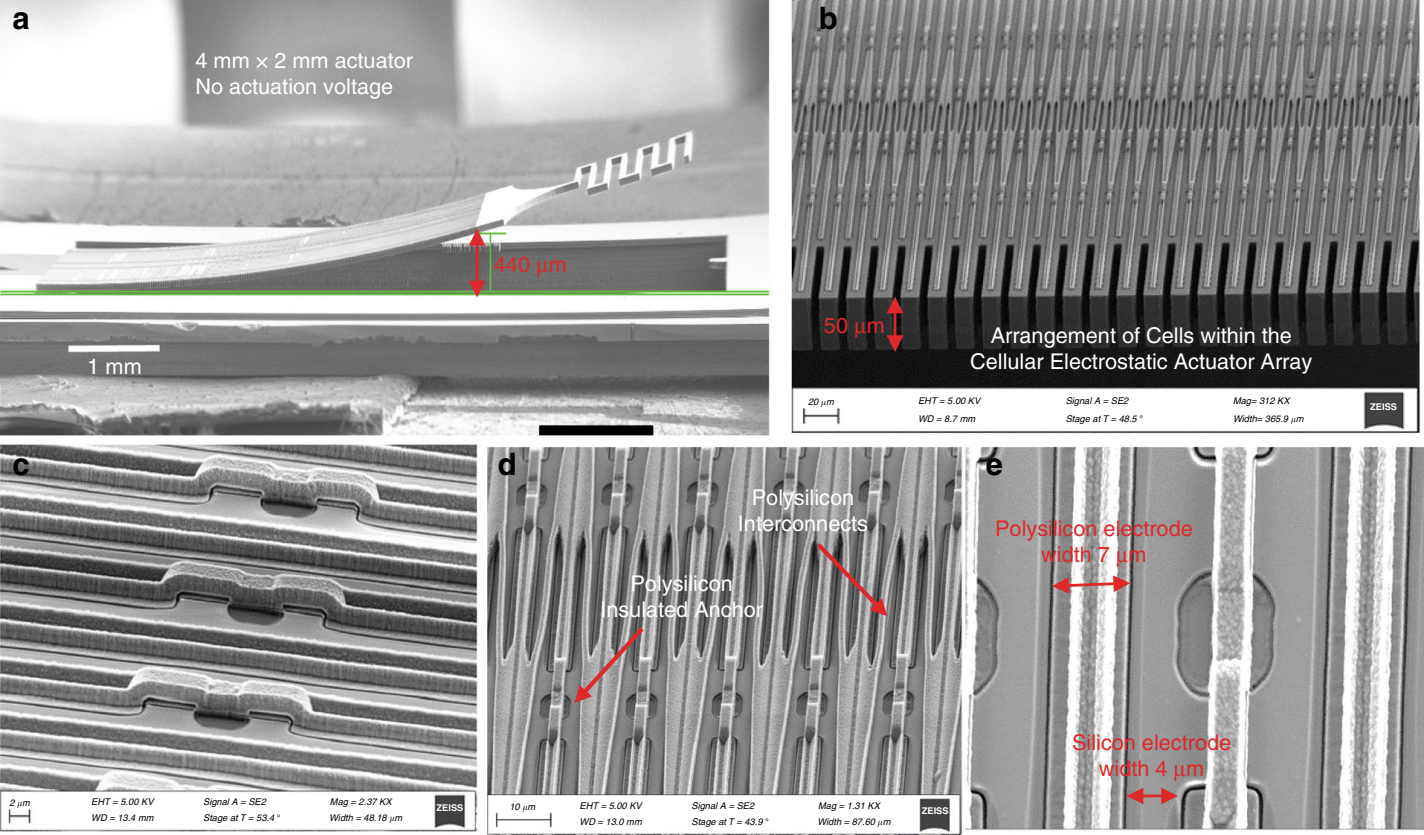
Structral design. **a** SEM image of a fabricated 4010 μm × 2010 μm × 50 μm cellular electrostatic actuator array with 7600 cells; (**b**) Zoomed in view of the brick wall arrangement of actuator cells (**c**) Zoomed in view of polysilicon electrodes and interconnects on the top surface of the device; (**d**) zoomed in view of polysilicon anchor points on the actuator silicon frame; (**e**) Zoomed in view of the air gap between polysilicon and the silicon frame sidewalls

Figure 2a shows a scanning electron microscope (SEM) view of a fabricated 4 × 2 mm actuator array consisting of 362 rows and 20 and 21 cells per row in alternative rows (a total of 7600 cells). Zoomed-in views of the flexible suspended polysilicon interconnects, their anchoring points to the silicon frame, and the air gaps are shown in Fig. 2b–e. Each cell is 95 µm long (trench length of 80 µm) and 11 µm wide (trench width of 7 µm). The transduction air gap between the crystalline silicon sidewalls and polysilicon electrodes is approximately 250 nm. Due to the residual stress in various deposited thin films forming the device, the array is curved upward with its tip raised above the surface without application of any actuation voltage.

**Fig. 3:**
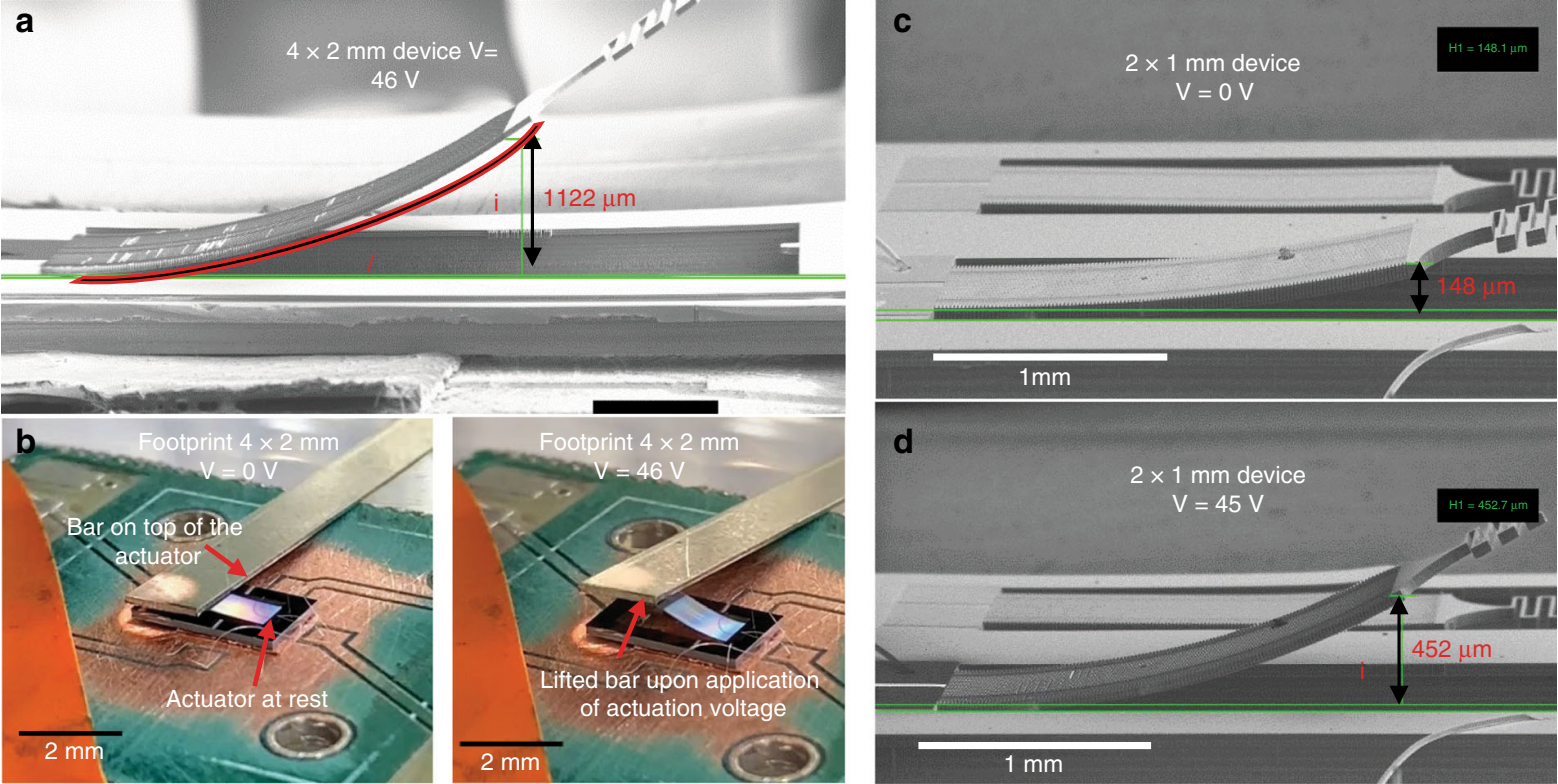
Characterization and performance validation. **a** SEM image of the actuator with footprint of 4 × 2 mm bent upwards upon the application of actuation voltage 46 V). **b** Shows the ability of device in lifting the end of a bar that is supported on a fulcrum with 46 V actuation voltage. **c**, **d** SEM image of 2 × 1 mm device before and after actuation

To characterize the performance of the actuators, DC tests were performed inside the SEM chamber with different DC actuation voltages applied between the silicon mesh and the polysilicon electrode network. As expected, the array curves further upward due to the electrostatic force between the silicon and polysilicon walls. Figure 3a shows the SEM view of the 4 × 2 mm actuator array of Fig. 2 with 46 V of actuation voltage being applied (maximum applied voltage recorded before the breakdown failure). The free end of the actuator array is ~1120 µm above the substrate surface (~680 µm vertical displacement compared to its resting position shown in Fig. 2a). Furthermore, a silicon chip with a mass of 8 mg was used to observe the weight lifting capability of the actuator. The supplementary video shows the actuator lifting the 8 mg weight, which is approximately 60× times heavier than the actuator itself. The weight is lifted by approximately 200 µm (visually estimated) with an actuation voltage of 45 V. Figure 3b also shows the ability of the device to lift the end of a bar that is supported on a fulcrum. Figure 3c, d show the SEM image of a 2 × 1 mm device before actuation and upon actuation at 45 V. The 2 × 1 mm array comprises 242 rows and 9 or 10 cells per row in alternative rows (total of 2299 cells) with individual cell sizes of 80 µm by 8 µm. A vertical deflection of up to 304 µm was measured for this device with 45 V of actuation voltage.

**Fig. 4:**
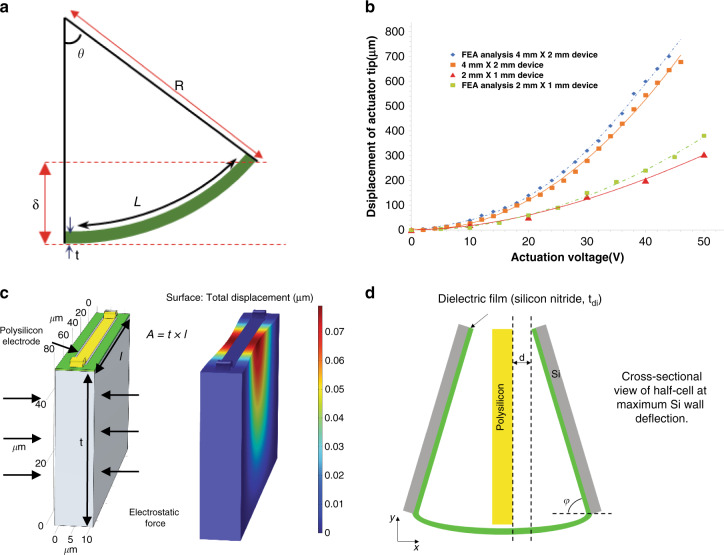
Analytical and FEA model analysis. **a** Schematic view of a deflected cantilever with critical dimensions of the curvature. **b** Graph presenting plot of displacement of the actuator upon increasing the actuation voltage for both 2 × 1 mm device and 4 × 2 mm device. FEA analysis result plot also shown, which complies with the experimental data. **c** Schematic of an individual cell and its displacement profile when force is applied along the sidewall of the cell. **d** Schematic view of the cross section of a cell with deflection of silicon sidewall

The radius of curvature of such a structure can be calculated based on the array dimensions and the displacement at its free end. The equation for the radius of curvature R for such a structure is:

1 $$R = \frac{L}{\theta }$$

2 $$\delta = \left( {1 - cos\theta } \right)R$$

where ***δ*** is the measured displacement of the actuator tip, *L* is the length of the actuator and ***θ*** is the arch angle of the device (Fig. 4a). The radius of curvature of such a bending beam for the 4 × 2 mm device upon the application of 46 V is calculated to be 11.8 mm (approximately 19° arch). To achieve such curvature, the array should have shrunk by 20 µm on its top surface, i.e., each of the air gaps in each cell should have shrunk by ~82 nm on the top (155 nm remaining air gap). This shows the leveraging of the displacement by the bending structure creating a much larger vertical displacement at the free end of the actuator (in the hundreds of microns range) from a few microns of total air gap change of the cells within the actuator (on the order of a few to tens of microns). Equation 3 relates the vertical displacement (***δ***) to the gap size change (*d*) within individual cells:

3 $${\it{\updelta }} = \frac{{{\it{nLd}}}}{{2{\it{t}}}}$$

where *n* is the number of cell rows, *L* is the length of the actuator and *t* is the thickness of the actuator.

The graph in Fig. 4b shows the measured displacement at the free end of the three different arrays with different actuation voltages. Finite element analysis results obtained from COMSOL electromechanics physics are also plotted on the graph of Fig. 4b, showing acceptable agreement between measurements and FEA given the many sources of potential error (e.g., dimensional inaccuracies, sidewall thickness variations along the trench thickness, built-in film stress, etc.). The force equation for parallel plate actuators with dielectric covered sidewalls is given by:

4 $${\it{F}}_{{\it{elec}},{\it{pp}}} = {\it{n}}\frac{{\varepsilon _0{\it{AV}}_{{\it{act}}}^2}}{{2\left( {{\it{g}} + \frac{{{\it{t}}_{{\it{di}}}}}{{\varepsilon _{\it{r}}}}} \right)^2}}$$

where *ɛ*_*0*_ is the permittivity of air (8.85 × 10^−12 ^F/m), *A* is the electrode area, *V*_*act*_ is the actuation voltage, g is the air gap width, t_di_, and *ɛ*_*r*_ are the dielectric thickness and relative permittivity, respectively, and *n* is the number of cells acting in each row. In the case of the designed actuators, there is a gradual change in the gap size along the cell from the top to the bottom (Fig. 4c). Considering this changing gap size, to estimate the force in each cell, the following equation is used:

5 $${\mathrm{F}} = {\int}_{\!\!0}^{{\boldsymbol{t}}} {\frac{{{\it{\upvarepsilon }}.{\it{l}}.{\it{y}}.{\it{V}}_{{\it{act}}}^2}}{{2({\it{d}} + {\it{y}}({\it{cotan\upvarphi }}))^2}}} {\it{dy}}$$

where *t* is the device thickness, *d* is the final air gap, *l* is the length of the electrode, *y* is the width of the electrode, and ***φ*** is the bending angle between the polysilicon electrode and the bent silicon wall with respect to the y-axis, as shown in Fig. 4d. The electrostatic force acting on the sidewalls of each cell is calculated to be approximately 1.5 mN with an actuation voltage of 46 V.

**Fig. 5:**
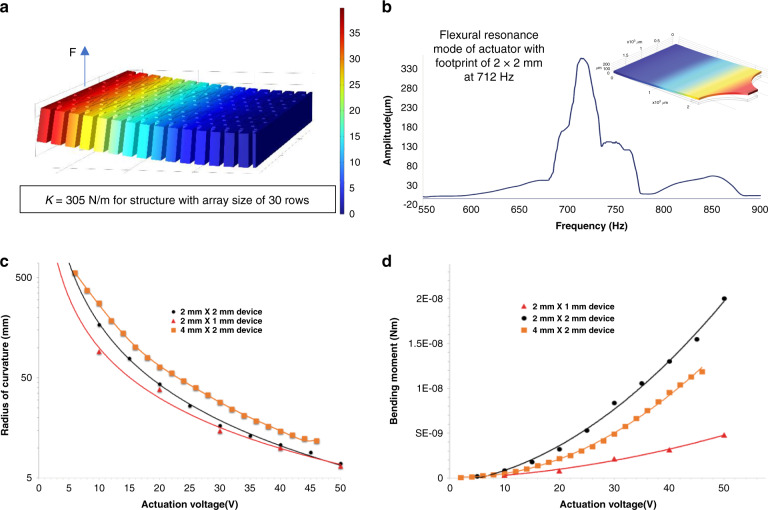
Static and dynamic performance of actuators. **a** Finite element analysis displacement profile for array structure with 30 rows of cells. Force has been applied at the end of the structure, and based on the displacement, the stiffness is calculated. **b** Frequency response of a 2 × 2 mm device showing resonance frequency at 712 Hz. **c** Graph presenting plot of the experimental results of radius of curvature for the three different device sizes upon increasing the actuation voltage. **d** Graph presenting experimental results of the bending moment with change of the actuation voltage for the three different actuator device sizes

To facilitate the analysis and predict the mechanical specifications of actuator designs with different dimensions, the effective Young**’**s modulus is defined for the actuator arrays. The effective Young**’**s modulus is the Young**’**s modulus required for a structural material forming a solid cantilever with the same dimensions as the actuator array to have the same mechanical stiffness as that of the actuator array. To estimate the effective Young**’**s modulus of such a complex structure, FEA using COMSOL solid mechanic physics was performed. Due to the physical memory limitations of the available PC, three different 3-D models of the actuator array (with cell dimensions the same as the actual devices with different array sizes), including both the silicon frame and polysilicon electrode network, were created in COMSOL, as shown in Fig. 5a. The flexural stiffness of each 3-D model of the actuator structure was found to be 8724 N/m, 1024 N/m, and 305 N/m for structures with array sizes of 10, 20, and 30 rows, respectively, at the free end of the array by applying a point load to the free end determining the resulting displacement. This stiffness was then compared to the stiffness of a solid monolithic clamp-free cantilever with similar dimensions using the following equation:

6 $${\it{k}} = \frac{{3{\it{E}}_{{\it{eff}}}{\it{I}}}}{{{\it{l}}^3}}$$

In this manner, the effective Young’s modulus of the composite structure is calculated to be 675 MPa for the 2 × 2 mm device.

Since the cell dimensions, including the width of silicon sidewalls, height, and length of each cell, determine the effective Young’s modulus and therefore the overall stiffness of the actuator, the following empirical equation shows the dependence of the effective Young’s modulus on the cell dimensions.

7 $${\it{E}}_{{\it{eff}}} = \frac{{{\it{\upalpha W}}_{{\it{cell}}}^3}}{{{\it{L}}_{{\it{cell}}}{\it{t}}^3}}$$

where *W*_*cell*_ is the width of the silicon sidewalls, *L*_*cell*_ is the length of each cell, *t* is the thickness of the actuator (height of each cell), and α is a coefficient (105 MN/m), which is extracted from the cell dimensions of the presented devices and the extracted effective Young’s modulus.

The calculated effective Young’s modulus also showed a very good fit with the flexural resonance frequency of the device (712 Hz), where the flexural resonance frequency of the composite structure is equal to the square root of the ratio of the flexural stiffness and mass of the actuator. Figure 5d shows the frequency response of the composite structure (the actuator with a footprint of 2 × 2 mm) around the vicinity of the resonance frequency of the device. To study the frequency response of the actuator, an AC signal with a predetermined DC offset excited the actuator, and the amplitude of the vibration was plotted in the frequency domain.

The bending moment of the curved structure due to electrostatic force acting between the silicon mesh and the polysilicon network can be obtained from the following equation:

8 $$M = \frac{{E_{eff}I}}{R}$$

where *I* is the area moment of inertia, *E*_*eff*_ is the effective Young’s modulus of the fabricated device, and *R* is the radius of curvature.

Knowing the effective Young’s modulus and the radius of curvature obtained using measurements and Eqs. (1) and (2), the bending moment for the tested device (4 × 2 mm device) was calculated to be 1.1 µNm with 46 V of actuation.

To find the energy output of the actuator, the following equation can be used:

9 $${\it{U}} = \frac{{{\it{M\uptheta }}}}{2}$$

where *M* is the bending moment and *θ* is the arch angle (19° in this case). This leads to a total output energy of 0.19 µJ for the tested actuator, which translates to an energy density per volume (total occupied volume including electric field and electrodes except for the supporting tethers) of 0.47 mJ/cm^3^. Figure 5c shows the graph for the radius of curvature versus the actuation voltage, and Fig. 5d shows the bending moment versus the actuation voltage for the three tested prototypes with different cell dimensions and overall footprints. The 2 × 2 mm^2^ actuator array exhibited up to 332 µm vertical displacement with 60 V actuation voltage. This device provides an energy density of 1.42 mJ/cm^3^, which is the highest value measured among the three prototypes tested.

Table 1 summarizes the specifications and test results for the three different tested actuators.

**Table 1:** Dimensions and performance metrics of three different cellular electrostatic actuators based on measurements, calculations, and finite element analysis

Device Footprint	2 × 1 mm	2 × 2 mm	4 × 2 mm
Cell length	80 µm	80 µm	80 µm
Silicon sidewall width	3 µm	4 µm	4 µm
Polysilicon electrode width	5 µm	7 µm	7 µm
Device layer thickness	50 µm	50 µm	50 µm
Vertical displacement upon actuation voltage	304 µm	333 µm	678 µm
Actuation Voltage*	45 V	60 V	46 V
Flexural stiffness of device	3.4 N/m	14.2 N/m	1.8 N/m
Effective Young’s modulus	301 MPa	675 MPa	675 MPa
Bending moment	4.7 × 10^−7^ Nm	2.0 × 10^−6^ Nm	1.1 × 10^−6^ Nm
Curvature (R^−1^)	0.18 mm^−1^	0.14 mm^−1^	0.08 mm^−1^
Energy Density	0.7 mJ/cm^3^	1.42 mJ/cm^3^	0.47 mJ/cm^3^
Normalized Bending moment**	2.32 × 10^−10^ Nm/V^2^	5.5 × 10^−10^ Nm/V^2^	5.48 × 10^−10^ Nm/V^2^
Normalized Curvature (R^−1^)**	0.09 m^−^1 V^−2^	0.04 m^−1^V^−2^	0.04 m^−1^V^−2^
Energy density***	1.7 × 10^−10^ J/cm^3^V^4^	1.05 × 10^−10^ J/cm^3^V^4^	1.05 × 10^−10^ J/cm^3^V^4^

*Maximum voltage at which the device had a breakdown failure.

**Normalized per actuation voltage squared.

***Normalized per total occupied volume including electric field and electrodes.

## Discussion

The displacement range of the movable electrode in a parallel plate electrostatic actuator is limited to the transduction gap between the electrodes. The presented cellular actuators are able to bypass this limitation and allow the realization of electrostatic actuators with both high energy density (submicron transduction gaps) and large displacements. This reinforces the idea of cascading individual cells to form the arrayed cell structures. The fabricated actuator showed a maximum displacement of 678 µm and a maximum energy density of 1.42 mJ/cm^3^. Table 2 shows a summary of the performance of the tested devices compared to some of the existing relevant work in the literature showing significantly higher energy density, displacement, and output force with a relatively low actuation voltage.

**Table 2:** Force, displacement, energy density, and actuation voltage of the arrayed cellular actuator compared to other works recently reported in this area of research

	Force	Displacement	Energy Density	Actuation voltage
Electrostatic piston tube actuator^44^	59 µN	28 µm	0.178 µJ/cm^3^*	80 V
Zipper microstate actuator^51^	32 µN	212 µm	NR*	135 V
Repulsive actuator for large out-of-plane force^52^	40 µN	15 µm	NR**	120 V
PZT actuator with MEMS enabled motion amplifier^53^	5.3 mN	3.3 µm	0.02 mJ/cm^3^ *	170 V
Nano electrostatic drive (NED) actuator^45^	NR**	226 nm	NR**	45 V
Piezoelectrically driven microactuator^54^	NR**	145 µm	NR**	22 V
Electrostatic MEMS repulsive comb-drive actuator^55^	NR**	58 µm	NR**	25 V
Shape memory alloy actuator for silicon microgrippers^25^	1150 µN	800 µm	NR**	NR**
Arrayed cellular electrostatic actuator	80 mN	678 µm	1.42 mJ/cm^3^	46 V

*Calculated by the authors from provided information.

**Not reported.

The maximum achievable force, displacement, curvature, and bending moment of an actuator ribbon are proportional to the square of the maximum actuation voltage that can be safely applied to the device without electrical breakdown. Furthermore, the energy density is proportional to the product of output force and displacement and is therefore proportional to the fourth power of the actuation voltage (V^4^). The energy density normalized to the fourth power of the actuation voltage for the 4 × 2 mm^2^ device is 1.05 × 10^−10 ^J/cm^3^V^4,^ which is also similar to the energy density normalized to the fourth power of the actuation voltage for the 2 × 2 mm^2^ device (1.09 × 10^−10 ^J/cm^3^V^4^).

The bending moment and curvature of the device are functions of the dimensions of the cross section and the actuation voltage squared. For example, the curvature and bending moment normalized per actuation voltage squared are 0.04 m^−1^V^−2^ and 5.48 × 10^−10^ Nm/V^2^ for the 4 × 2 mm^2^ device, respectively, which is similar to the curvature and bending moment normalized per square voltage of the 2 × 2 mm^2^ device (0.04 m^−1^V^−2^, 5.5 × 10^−10^ Nm/V^2^), which has similar cell dimensions.

Electrostatic pull-in, which is a normal behavior of parallel-plate electrostatic actuators, has not been observed in any of the presented measurements. This is mainly due to the electrical breakdown occurring before reaching the pull-in voltage and observing the large sudden displacement of the actuator all the way to the point of full closure of the air gaps on top.

With a 500 nm thick silicon nitride film as the dielectric (*ɛ*_*r*_ *=* *8*) and an air gap of 500 nm, the maximum estimated actuation voltage is supposed to be approximately 140 V, while the fabricated devices exhibit breakdown voltages below 60 V during testing. There are several hypotheses that can be the cause of this early breakdown. One reason can be the non-conformality of LPCVD nitride film deposition, which could result in the thinner nitride film at the bottom of the trenches. Alongside that, the relatively long wet etching process of the sacrificial oxide (20–25 min) could thin down the dielectric film (nitride) further, which might also result in lowering of the breakdown voltage even though the selectivity wet etch of the oxide to nitride in 49% HF is high (1:250).

## Conclusion

Micromachined electrostatic cellular actuators with submicron high aspect ratio transduction air gaps were fabricated and characterized. A vertical displacement of approximately 678 µm for a 4 × 2 mm^2^ device at 46 V and an energy density of 1.42 mJ/cm^3^ were measured for a 2 × 2 mm^2^ actuator. Early breakdown of the wire bond pads is an issue that needs to be further studied. The long-term reliability and durability test study provides more insight into further improving the performance of the actuator. Prolonged durability of the actuator components (especially the sidewall dielectric film) under millions to billions of full actuation cycles with potential physical contact between the sidewalls would provide insight into the mechanical viability of the actuator. Endurance of the giant electric field between the narrow air gaps and the dielectric films covering the gap sidewalls over the long lifetime of the actuator and millions of operating cycles is also an important factor for improving further performance output of the designed actuator.

## Materials and methods

### Actuator fabrication

**Fig. 6:**
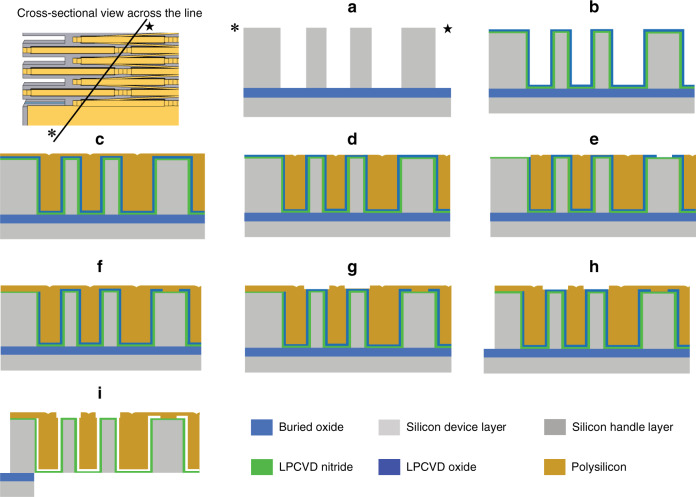
Fabrication process flow diagram. Schematic showing cross-sectional view of the modified HARPSS micromachining process flow used for fabrication of the high-output cellular electrostatic actuator arrays

Figure 6 shows the cross-sectional schematic view of the process flow used for fabrication of the described cellular actuator arrays on silicon on insulator (SOI) substrates (device layer resistivity of 0.005 Ω.cm) using the modified version of the high aspect ratio polysilicon and silicon (HARPSS) fabrication process^48^. The process starts by deep reactive ion etching (DRIE) of vertical trenches into the silicon device layer (50 µm thick) of the SOI substrate. Trenches extend all the way through the device layer to the SOI buried oxide layer (BOX of 2 µm) (Fig. 6a). This step defines the crystalline silicon mesh of the actuators keeping the silicon device layer around the actuator intact (the mesh is still part of the surrounding device layer). A thermal oxidation and oxide removal step is then performed to remove the surface roughness (scalloping and striations) induced on the silicon sidewalls during deep silicon etch to form the trench. A conformal layer low stress silicon nitride of 500 nm is then deposited via LPCVD covering the sidewalls. This is followed by a 250 nm thick layer of conformal silicon dioxide deposited via LPCVD, which serves as the sacrificial layer defining the transduction air gap between the crystalline silicon sidewalls and polysilicon electrodes (Fig. 6b). A 3.5 µm thick layer of LPCVD p-doped polysilicon is then deposited to refill the trenches and form the vertical electrodes within the cells (Fig. 6c). Polysilicon is then blanket-etched on the top surface (Fig. 6d), providing access to the underlying sacrificial silicon oxide layer. The 2nd lithography step is then performed to selectively remove the oxide film from certain areas where the electrodes are to be anchored onto the silicon mesh (Fig. 6e). A second layer of p-doped LPCVD polysilicon (1.5 µm thick) is then deposited (Fig. 6f). After this, annealing at 1100 degrees is performed, which improves the conductivity of the polysilicon. The 2nd polysilicon is then patterned (the 3rd lithography step), forming interconnections between the polysilicon electrodes and electrode anchors to the silicon frame (at nodal points where deformation of the frame is close to zero). The underlying nitride layer provides electrical isolation between the anchored polysilicon and silicon mesh. Processing continues by plasma etching of the second polysilicon layer (Fig. 6g). Another topside lithography step is then performed to pattern the silicon device layer around the actuator arrays defining the outline of the devices as well as forming the supporting tethers (Fig. 6h). The actuators have relatively large sizes in the few-millimeter range; therefore, to avoid stiction and minimize the time required to undercut the silicon structures (during BOX layer removal at the end of the process), the silicon handle layer underneath the arrays is removed. For this purpose, a backside lithography step followed by a long through handle layer DRIE is performed. Finally, the sacrificial oxide between the nitride-covered silicon and polysilicon sidewalls within the deep trenches is removed by a 20-minute-long dip in 49% hydrofluoric acid (HF) solution (Fig. 6i). Silicon nitride has a very low etch rate in HF, and therefore, most of the thickness of the nitride layer covering the silicon sidewalls is expected to remain in place.

## Supplementary information

Table 1-2

Test inside SEM chamber #2

Actuation with out load

Actuation with Load #1

Actuation with Load #2

Test inside SEM chamber # 1
