# Supplementary material for: Cellular shape micromachined actuator ribbons
Source: Microsyst Nanoeng. 2022 Aug 3;8:87. doi: 10.1038/s41378-022-00421-y (PMC9345908; doi:10.1038/s41378-022-00421-y)
Supplement: Supplementary file 1 — Table 1-2 [file 41378_2022_421_MOESM1_ESM.pdf]

Table 1. Dimensions and performance metrics of three different cellular electrostatic actuators based on measurements, calculations, and finite element analysis.

**Device Footprint**	**2×1 mm**	**2×2 mm**	**4×2 mm**
Cell length	80 µm	80 µm	80 µm
Silicon sidewall width	3 µm	4 µm	4 µm
Polysilicon electrode width	5 µm	7 µm	7 µm
Device layer thickness	50 µm	50 µm	50 µm
Vertical displacement upon actuation voltage	304 µm	333 µm	678 µm
Actuation Voltage*	45 V	60 V	46 V
Flexural stiffness of device	3.4N/m	14.2 N/m	1.8 N/m
Effective Young's modulus	301 MPa	675 MPa	675 MPa
Bending moment	4.7×10^{-7}Nm	2.0×10^{-6} Nm	1.1×10^{-6} Nm
Curvature (R^{-1})	0.18 mm^{-1}	0.14 mm^{-1}	0.08 mm^{-1}
Energy Density	0.7 mJ/cm^3	1.42 mJ/cm^3	0.47 mJ/cm^3
Normalized Bending moment**	$2.32\times 10^{-10}$$Nm/V^2$	5.5×10^{-10} Nm/V^2	5.48×10^{-10} Nm/V^2
Normalized Curvature (R^{-1}) **	0.09 $m^{-}1$ V^{-2}	0.04 $m^{-1}V^{-2}$	0.04 $m^{-1}V^{-2}$
Energy density ***	1.7×10^{-10} J/cm^3V^4	1.05×10^{-10} J/cm^3V^4	1.05×10^{-10} J/cm^3V^4

* *Maximum voltage at which the device had a breakdown failure*

** *Normalized per actuation voltage squared.*

*** *Normalized per total occupied volume including electric field and electrodes.*

Table 2. Force, displacement, energy density, and actuation voltage of the arrayed cellular actuator compared to other works recently reported in this area of research.

	Force	Displacement	Energy Density	Actuation voltage
Electrostatic piston tube actuator [44]	59 µN	28 µm	0.178µJ/cm^3 *	80 V
Zipper microstate actuator [51]	32 µN	212 µm	NR *	135 V
Repulsive actuator for large out-of-plane force [52]	40 µN	15 µm	NR **	120 V
PZT actuator with MEMS enabled motion amplifier [55]	5.3 mN	3.3 µm	0.02 mJ/cm^3 *	170 V
Nano electrostatic drive (NED) actuator [45]	NR **	226 nm	NR **	45 V
Piezoelectrically driven microactuator[53]	NR **	145 µm	NR**	22 V
Electrostatic MEMS repulsive comb-drive actuator [54]	NR**	58 µm	NR**	25 V
Shape memory alloy actuator for silicon microgrippers[25]	1150 µN	800 µm	NR**	NR**
**Arrayed cellular electrostatic actuator**	**80 mN**	**678 µm**	**1.42 mJ/cm3**	**46 V**

** Calculated by the authors from provided information*
*** Not reported*
